# A New and Original Curiosity of Medical Literature

**Published:** 1847-02

**Authors:** 


*A New and Original Curiosity of Medical Literature.*—Wonders will never cease. Who could have thought some time ago that a "celebrated obstetric physician" in the metropolis could treat a lady in the country by electrical telegraph, yet to that complexion has it come.—*Ibid.*

"*Consultation per Telegraph.*—The services of the electric telegraph between Norwich and Shoreditch were put into requisition on Thursday in a novel manner, being made the means of communication between a physician in London and his patient in the former place. On Wednesday Dr. L., *a celebrated obstetric physician*, was sent for from London to attend a lady, lying there in a dangerous state; on his return to town, he left instructions to the medical attendant to convey information of the state of the patient the next morning by means of the telegraph. This was promptly done on Thursday morning, and the prescription was as promptly returned. This it would appear, was repeated more than once, the services of the telegraph being continued for four hours. Unhappily the telegraph completed its communication by announcing the *death* of the lady." *Essex Herald.*

